# Non-invasive Fibrosis Markers for Predicting Esophageal Varices: A Potential Alternative to Endoscopic Screening

**DOI:** 10.7759/cureus.56433

**Published:** 2024-03-19

**Authors:** Kunza Ali, Saad Slah-Ud-Din, Mishal Afzal, Mah R Tariq, Tallha Waheed, Haroon Yousuf

**Affiliations:** 1 Medicine and Surgery, Shalamar Medical & Dental College, Lahore, PAK; 2 Internal Medicine, Shalamar Medical & Dental College, Lahore, PAK; 3 Internal Medicine, Jinnah Hospital, Lahore, PAK; 4 Graduate Medical Education, Combined Military Hospital (CMH) Lahore Medical College and Institute of Dentistry, Lahore, PAK; 5 Internal Medicine, Combined Military Hospital (CMH) Lahore Medical College and Institute of Dentistry, Lahore, PAK; 6 Gastroenterology, Shalamar Hospital, Lahore, PAK

**Keywords:** non-invasive markers of fibrosis, endoscopy, screening, liver injury, fibrosis markers, esophageal varices

## Abstract

Background: Chronic liver infections and diseases lead to chronic liver injury, which results in fibrosis. Due to this continuous scarring and regeneration, cirrhosis occurs, which is also responsible for several adverse sequelae, including but not limited to esophageal varices. Cirrhosis has resulted in patients' increased morbidity and mortality, especially in low socioeconomic settings such as Pakistan. Endoscopy is the gold standard for measuring the presence or absence of esophageal varices, along with their grade. Currently, some non-invasive markers (aspartate aminotransferase-to-alanine aminotransferase ratio (AAR), fibrosis 4 (FIB-4), AAR to platelet ratio index (AARPRI), aspartate aminotransferase-to-platelet ratio index (APRI), S-index, King’s score) are being established that make use of laboratory tests, such as a complete blood profile, liver function profile, and coagulation profile, to estimate the extent of hepatic fibrosis.

Objectives: The objective of this study is to establish a correlation between non-invasive markers of fibrosis and the presence of esophageal varices and to assess their potential as a substitute for gastrointestinal endoscopy screening. Additionally, the study aims to compare these six scores, thereby generating data on their individual and relative accuracy.

Methodology: This was a cross-sectional study conducted at the Shalamar Institute of Health Sciences, Lahore, Pakistan. Outpatient (OPD) data were obtained from the Shalamar online portal system from June 2022 to December 2022. Laboratory tests, abdominal ultrasounds, and endoscopy results were accessed and recorded in the questionnaire. The patient’s medical records and contact numbers were also noted in case further questions arose. Data were then compiled into a Microsoft Excel spreadsheet (Microsoft Corp., Redmond, WA) and analyzed after computing the non-invasive procedure formulas. It was analyzed using IBM SPSS Statistics for Windows, version 20.0 (IBM Corp., Armonk, NY). P-values were calculated, and conclusions were drawn.

Results: Of the sample size of 100 patients with liver damage and injury, 60% were male and 40% were female. Among males, 15% had a milder (grade 1) degree of esophageal varices, and 45% had a moderate to advanced degree (grades 2-3) of esophageal varices. Among females, 19% had mild (grade 1) varices, while 21% had severe (grade 3) varices. The most common cause of varices in patients who had developed fibrosis and/or cirrhosis was hepatitis C, with a wide margin of other causes. The p-values obtained showed that from the selected list of non-invasive markers of fibrosis, only FIB-4 and AARPRI were statistically significant with p-values of 0.036 and 0.022, respectively.

Practical implications: Though endoscopy is currently the gold-standard procedure for detecting the presence or absence and grade of esophageal varices, it is invasive, which makes the patients extremely uncomfortable and apprehensive. It can also lead to post-procedure infection, internal hemorrhages, and trauma due to instrument use. Due to its invasive nature, some patients also tend to refuse this procedure. Non-invasive fibrosis markers can help make a diagnosis without undergoing an endoscopy, which in turn will improve patient compliance and satisfaction.

Conclusion: It was observed that FIB-4 and AARPRI can be used together as reliable markers to assess the presence or absence of esophageal varices.

## Introduction

Cirrhosis is defined as the scarring of hepatic tissue due to persistent tissue injury attributed to multiple causes. Histologically, it is characterized by the formation of simultaneous fibrous bands and regenerative nodules as a result of chronic insult to the liver [1]. Cirrhosis has two main stages: compensated and decompensated with distinct presentations and prognoses. The compensated stage is asymptomatic, with a median survival of more than 12 years, and it is at this stage that the diagnosis is difficult and requires a high index of suspicion. The decompensated stage includes patients who have obvious clinical complications of cirrhosis, such as variceal hemorrhage (VH), ascites, and/or hepatic encephalopathy, with a median survival of less than two years. If using the Child-Turcotte-Pugh (CTP) classification, patients in the CTP-A class are compensated, and those in the CTP-B/C class are mostly decompensated. Cirrhosis results in several sequelae, which complicate the disease further, resulting in severe morbidity and even mortality [2]. One major complication is portal hypertension, which is mainly caused by blood flow obstruction in the liver itself due to its distorted, coarse architecture [3]. Collaterals are formed, which shunt blood from the portal circulation to the systemic circulation [4]. This results in the development of esophageal varices of variable grades, which increase the risk of rupture and bleeding [5,6]. In compensated cirrhosis, the prevalence of any variceal bleed is 30%-40%, and only a minority (10%-20%) have high-risk varices (HRV) requiring treatment. These are medium or large varices or small varices with red wale marks, associated with an approximately 15% risk of bleeding per year. About 33% of the cirrhotic population with esophageal varices bleeds, and approximately two-thirds of that percentage experience recurrent variceal bleeding episodes [7]. Screening for varices must be done as soon as the patient is diagnosed with a cirrhotic liver to exclude any sinister events.

Nowadays, several invasive and non-invasive screening methods are available [8,9]. With its high sensitivity and specificity, esophagogastroduodenoscopy (EGD) is the benchmark for detecting variceal bleeding. However, its non-availability in distant areas, high cost, invasiveness, and the discomfort patients experience during the procedure are a few of the most common demerits. This in turn has resulted in the popularity of less intrusive procedures for variceal bleeding detection, which are not as accurate as EGD but are more favored by the population in general [10,11].

Fibrosis-4 (FIB-4), aspartate aminotransferase-to-alanine aminotransferase ratio (AAR), aspartate aminotransferase-to-platelet ratio index (APRI), S-index, AAR to platelet ratio index (AARPRI), and King’s score indexes are some of the non-invasive scores for a relative estimation of liver scarring or fibrosis [11,12]. They include the parameters of the patient’s age, aspartate aminotransferase (AST), alanine aminotransferase (ALT), gamma-glutamyl transpeptidase (GGT), albumin, and platelet count (PLT). Laboratory tests that include these parameters are routinely completed to check liver function and obtain complete blood analyses, making these parameters easily available for use and consequent documentation [13,14].

The objective of this study is to correlate non-invasive markers with the presence of esophageal varices observed during EGD and assess their potential as a substitute for gastrointestinal endoscopy screening. Additionally, the study aims to compare these six scores, thereby generating data on their individual and relative accuracy.

## Materials and methods

This was a cross-sectional study conducted at the Shalamar Institute of Health Sciences, Lahore, Pakistan, after receiving ethical approval from Shalamar Medical & Dental College's Institutional Review Board (approval number: SMDC-IRB/AL/14/2022). Outpatient (OPD) files were obtained from the Shalamar online portal system from June 2022 to December 2022 after obtaining permission from the respective gastroenterology department consultants and maintaining patient confidentiality by not disclosing their names or identities. The laboratory tests (such as ALT, AST, GGT, and international normalized ratio (INR)), abdominal ultrasounds, and endoscopy results indicating the severity of esophageal varices, along with the patient’s demographic details, were accessed and recorded on the questionnaire. The questionnaire was adapted for this research study by the study's authors themselves (Appendix A). Additional knowledge was also added and recorded to pave the way for future studies with similar backgrounds.

The inclusion criteria were all the patients who came to the OPD between the ages of eight and 80 with records of all the above labs, imaging, and endoscopic findings. Patients who had not undergone endoscopy or were without the complete medical records needed to fulfill this study’s criteria on the online portal were excluded from the study. Patients under the age of eight or over the age of 80 were also excluded from the study. Only the patients who had coarse echotexture on their ultrasound findings were included in this research. The patient’s medical records and contact numbers were also noted in case further questions arose. Information from 100 patients was recorded on the self-administered and adapted questionnaire. Some patients were also contacted via telephonic correspondence if the required information was ambiguous or absent from the questionnaire. It was ensured that the Health Insurance Portability and Accountability Act (HIPAA) laws were adhered to by making sure that patients' names and identities were never disclosed; instead, an arbitrary number was allocated to each data collection form. Data were then added to a Microsoft Excel spreadsheet (Microsoft Corp., Redmond, WA) and analyzed after computing the non-invasive procedure formulas. The formulas are as follows: 

1. AAR = AST/AL2. FIB-4 = (Age in yrs. × AST)/(PLT × √ALT)

3. APRI = (AST/normal upper limit) × 100/PLT (109/L)

4. AARPRI = AAR/PLT count (109/L)/150 

5. S-index = 1000 × GGT/(PLT × albumin2) 

6. King’s score = Age × AST × INR/PLT [12,15].

The main aim was to screen for the presence of esophageal varices and determine their severity. This was done by endoscopy. Esophageal varices are classified according to the standard nomenclature, as outlined as follows: grade 1 (small) where the esophageal varices are straight and small; grade 2 (moderate) where the varicose veins are relatively large in size, stringy in shape, and occupy an area of more than one-third in the lumen of the esophagus and when inflated, the dilatations do not collapse; and grade 3 (large) where the varicose veins are large in size, shaped like a tumor, and occupy an area of more than 1⁄3 in the lumen of the esophagus.

The Child-Pugh scoring system also referred to as the Child-Pugh-Turcotte score, was then used to determine the severity of hepatic cirrhosis. It comprises three classifications: class A indicates good hepatic function, class B indicates moderately impaired hepatic function, and class C indicates advanced hepatic dysfunction. Originally, this scoring system utilized five clinical and laboratory parameters for patient categorization: serum bilirubin, serum albumin, ascites, neurological status, and clinical nutrition.

The scoring system was modified later by Pugh et al., substituting prothrombin time (PT) for clinical nutrition status. Additionally, they introduced variable points for each criterion based on increasing severity which were as follows: Encephalopathy: none = 1 point; grades 1 and 2 = 2 points; grades 3 and 4 = 3 points; Ascites: none = 1 point, slight = 2 points, moderate = 3 points; Bilirubin: under 2 mg/ml = 1 point; 2 to 3 mg/ml = 2 points; over 3 mg/ml = 3 points; Albumin: greater than 3.5 mg/ml = 1 point; 2.8 to 3.5 mg/ml = 2 points; less than 2.8 mg/ml = 3 points; Prothrombin time* (sec prolonged): less than 4 sec = 1 point, 4 to 6 sec = 2 points, over 6 sec = 3 points

*Frequently, INR will be used as a substitute for PT, with INR under 1.7 = 1 point, INR 1.7 to 2.2 = 2 points, and INR above 2.2 = 3 points.

The severity of cirrhosis was as follows: Child-Pugh class A: 5 to 6 points; Child-Pugh class B: 7 to 9 points; Child-Pugh class C: 10 to 15 points

The statistical analysis of the data was carried out using IBM SPSS Statistics for Windows, version 20.0 (IBM Corp., Armonk, NY). The mean ± SD was used for quantitative variables, while numbers and % were used for qualitative data. To assess differences in means of quantitative variables, the independent samples t-test and one-way ANOVA test were applied. The correlation was analyzed using the Pearson correlation coefficient. The statistical methods were verified using a significant value of p <0.05 and a highly significant level of p <0.001. After this, conclusions and inferences were drawn and noted.

## Results

**Figure 1:**
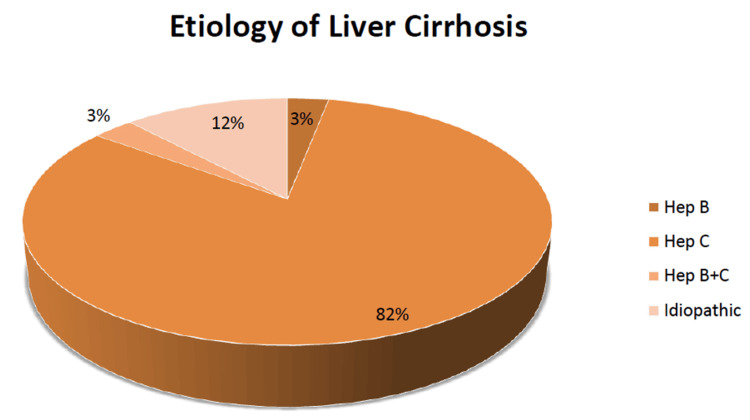
The etiology of liver cirrhosis in the study group Hep: hepatitis

The data of 100 patients were collected using online patient records. Only cases with established evidence of cirrhosis were included in the study. The most common cause of varices in patients who had developed fibrosis and/or cirrhosis was hepatitis C at 82%, with a wide margin of other causes. Furthermore, 12% of cases were idiopathic, followed by hepatitis B at 3%, and a combined pathology of hepatitis B and C was also at 3%. During this research study, there were no cases reported in the OPD of patients with metabolic dysfunction-associated steatotic liver disease (MASLD), alcoholic liver disease, or autoimmune hepatitis, probably due to the stigma around alcoholism and the lack of cost-effective and readily available screening methods in Pakistan for MASLD and autoimmunity (Figure 1).

**Figure 2:**
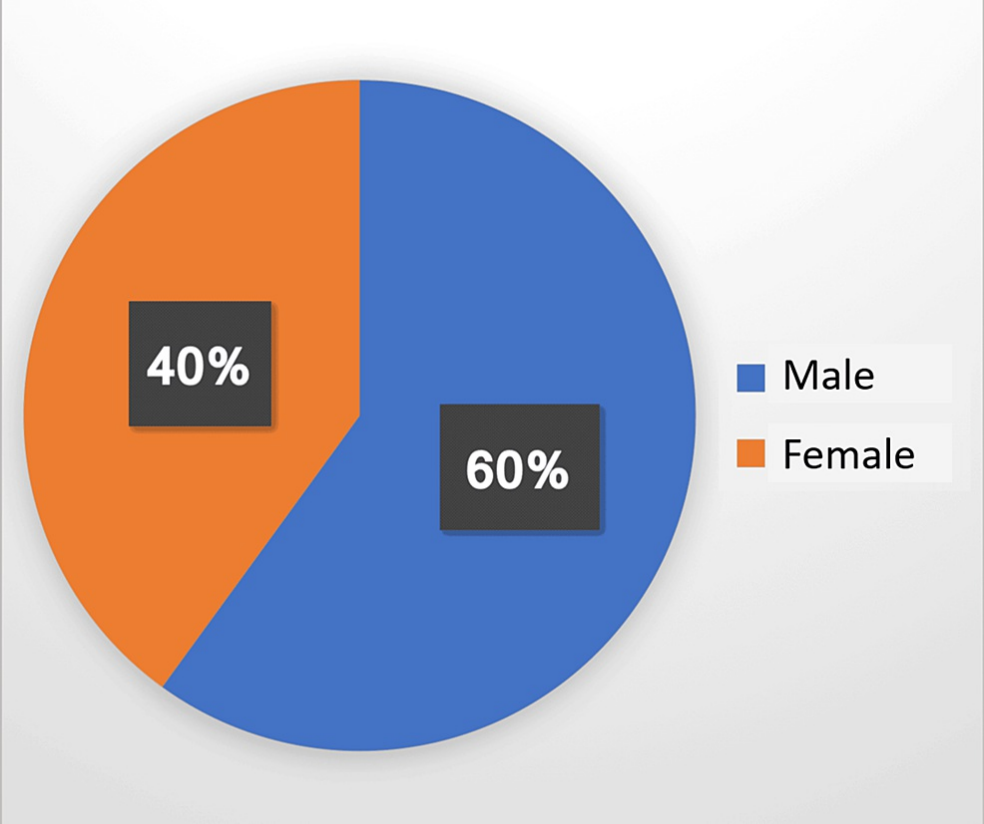
Gender-wise distribution of the sample with liver damage and injury

Of the sample size of 100 patients with liver damage and injury, 60% were male and 40% were female (Figure 2).

Among males, 15% had a milder (grade 1) degree of one or no esophageal varices, and 45% had a moderate (grade 2) to advanced degree of varices. Among females, 19% had mild (grade 1) varices, while 21% had severe (grade 3) varices. 

**Figure 3:**
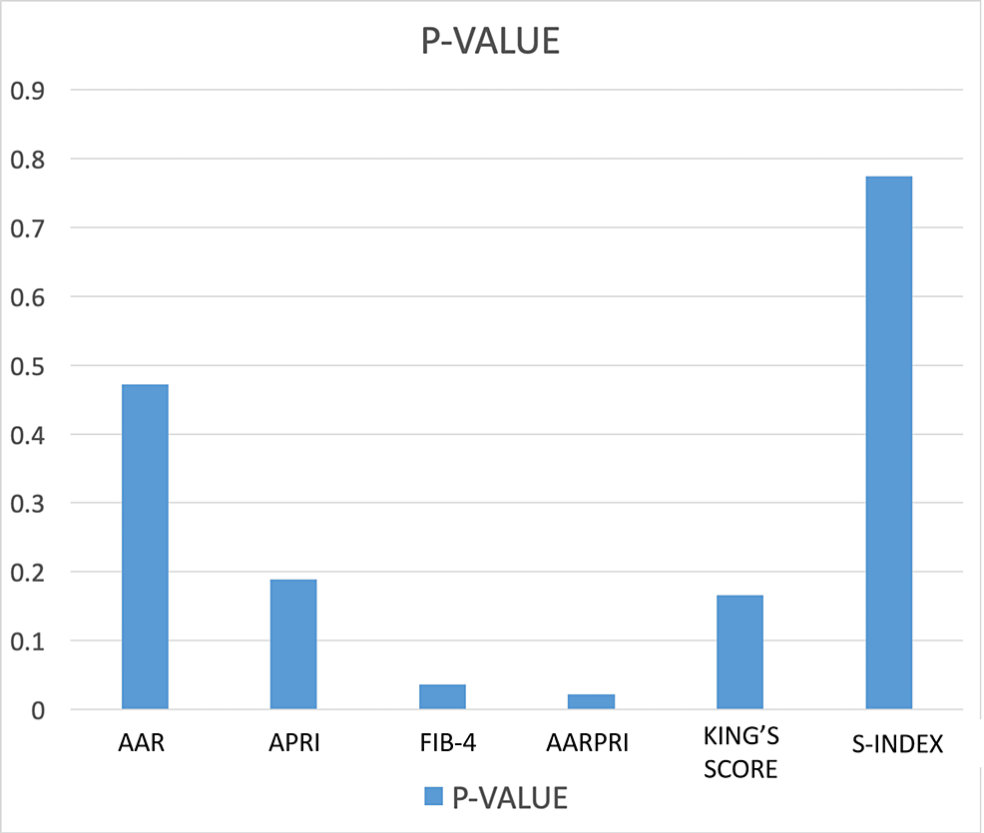
P-values obtained from the selected list of non-invasive fibrosis markers AAR: aspartate aminotransferase-to-alanine aminotransferase ratio; APRI: aspartate aminotransferase-to-platelet ratio index; FIB-4: fibrosis-4; AARPRI: AAR to platelet ratio

Overall, the cases of mild (grade 1) esophageal varices were 26% (26 cases), as seen on endoscopy. Moderate (grade 2) esophageal varices had the highest number of cases, with 44% (44 cases) of patients, and cases with severe (grade 3) varices were reported to be at 22% (22 cases). Patients with no varices made up 8% (eight cases) of the total patients. When the severity of hepatic fibrosis was calculated for each case using the Child-Pugh Classification (CPC), it displayed a significance of p = 0.046. Patients with moderate to severe esophageal varices tended to have a higher proportion of class B and class C CPC scores. Of the 71 reported cases of class B and class C CPC, 66 had moderate-severe (grades 2-3) esophageal varices on endoscopy. The data were then entered into IBM SPSS software. The p-values obtained showed that from the selected list of non-invasive markers of fibrosis, only FIB-4 and AARPRI were statistically significant with p-values of 0.036 and 0.022, respectively (Figure 3).

The APRI, S-index, AAR, and King's score had a p-value of 0.189, 0.774, 0.472, and 0.166, respectively, and they were not statistically significant enough to be used to draw comparisons or infer definite diagnoses and conclusions. The results are briefly summarized in Table 1.

**Table 1:** P-values obtained from the selected list of non-invasive fibrosis markers EV: esophageal varices; AAR: aspartate aminotransferase-to-alanine aminotransferase ratio; FIB-4: fibrosis-4; APRI: aspartate aminotransferase-to-platelet ratio index; AARPRI: AAR to platelet ratio

Variable		Total no. of patients (n=100)	No/mild EV (n=34)	Moderate- severe EV (n=66)	P-value
Gender distribution	Male	60	15 (44.1%)	45 (68.2%)	0.020
	Female	40	19 (55.9%)	21 (31.8%)	
Age (years)		3±1	3±1	3±0	0.213
Etiology (%)					0.031
Hepatitis B		3 (3%)	3 (8.8%)	0 (0%)	
Hepatitis C		82 (82.0%)	27 (79.4%)	55 (83.3%)	
Hepatitis B & C		3 (3%)	2 (5.9%)	1 (1.5%)	
Idiopathic		12 (12%)	2 (5.9%)	10 (15.2%)	
Varices, n (%)					<0.01
No		8 (8%)	8 (23.5%)	0 (0%)	
Mild		26 (26%)	26 (76.5%)	0 (0%)	
Moderate		44 (44%)	0 (0%)	44 (66.7%)	
Severe		22 (22%)	0 (0%)	22 (33.3%)	
Child-Pugh Classification (CPC), n (%)					0.57
Class A		29	15 (44.1%)	14 (21.2%)	
Class B		49	13 (38.2%)	36 (54.5%)	
Class C		22	6 (17.6%)	16 (24.2%)	
CPC		2±1	2±1	2±0	0.046
Fibrosis markers					
AAR		1.50±0.50	1.4±0.72	1.5±0.50	0.472
FIB-4		5.12±5.90	4.08±5.02	5.56±6.80	0.036
APRI		1.37±1.86	1.04±2.38	1.53±1.59	0.189
AARPRI		2.48±2.28	2.08±2.57	2.84±2.41	0.022
S-Index		61.23±96.38	52.19±147.6	74.04±83.83	0.774
King's Score		38.11±77.04	33.96±71.64	40.81±77.26	0.166

## Discussion

Liver cirrhosis, secondary to its many causes, imposes a significant burden on health resources worldwide. While alcoholism and chronic hepatitis C remain the leading etiologies in first- and third-world countries alike, our study highlighted hepatitis C as the leading pathology [16,17]. Even in the USA, a developed country, the total reported cases of cirrhosis are around 0.27%, and most of these individuals had no knowledge of the progression or prognosis of the disease due to the inaccessibility of affordable disease screenings [18].

Keeping that in view, when the results of our study were further divided based on disease severity among the selected cases, the majority of them had moderate-severe (grades 2-3) esophageal varices. When the severity of hepatic fibrosis was calculated for each case using CPC, it displayed marginal significance (p = 0.046). Patients with moderate to severe esophageal varices tended to have a higher proportion of class B and class C CPC scores. This implies a potential association between the severity of liver damage and the development of more serious esophageal varices.

A study conducted in different liver transplantation centers with 346 subjects found that, based on splenomegaly, platelet count, and physical examination, it was possible to divide the varices into two classes, i.e., high-risk of bleeding and low-risk of bleeding. These non-invasive markers corroborated the findings on endoscopy and proved less costly as well [19].

Today, clinicians are concerned with identifying some non-invasive biochemical markers with high sensitivity and specificity that are cheaper and easier to obtain to reduce the number of upper gastrointestinal endoscopies for screening and treating esophageal varices in liver patients. These non-invasive biomarkers are applied using routine laboratory tests that do not require extra cost, special devices, or additional biochemical tests. In the past, esophageal transection or distal splenorenal shunting, transportal obliteration, left gastric artery embolization, and partial splenic artery embolization were some of the common procedures used to treat bleeding esophageal varices [20]. Over time, endoscopic methods such as banding and ligation were employed to effectively eliminate varices. However, their cost-effectiveness was limited, as only 9%-36% of cirrhotic patients with large, bleeding esophageal varices underwent definitive treatment [21].

Likewise, another retrospective study of 229 patients completed in the liver clinic from July 2004 to August 2007 revealed that the Child-Pugh score of B/C, low platelet count, and spleen diameter can successfully predict the presence and a higher grade of esophageal varices [22].

According to a study, portal hypertension results in esophageal varices and is present in approximately 50% of patients with cirrhosis of the liver. The severity of esophageal varices directly corresponds with the grade of liver disease. Of patients who fall under Child-Pugh class C, 85% have varices, while only 45% of patients who fall under Child-Pugh class A suffer from varices [23].

Another study of 218 cirrhotic patients who had undergone screening endoscopy showed that the data obtained from platelet count and spleen diameter count directly corroborated with those of endoscopic findings and had an accuracy parameter of 86% [24].

A research study completed between January 2016 and January 2018 reported that 91 subjects with hepatitis C had their liver biopsies done and, afterward, had their AAR, FIB-4, Göteborg University Cirrhosis Index (GUCI), and King’s scores measured. They showed a positive correlation with the histological findings and had a significant value of less than 0.001 [25]. However, in another study, patients to whom antiviral therapy was administered did not show very efficacious results nor a correlation between FIB-4 and APRI with a degree of fibrosis regression after therapy [26].

Currently, studies are attempting to correlate the accuracy of non-invasive markers, such as FIB-4, APRI, AARPRI, AAR, S-index, and King’s score, with the endoscopic findings of varices. A meta-analysis done based on the International Prospective Register of Systematic Reviews (PROSPERO) database showed that APRI, AAR, FIB-4, Lok index, and Forns index were not able to successfully showcase nor correspond to the endoscopic findings of esophageal varices. Thus, these scores would not be acceptable replacements for invasive procedures such as endoscopy [27].

Another retrospective analysis completed with 650 subjects from January 2012 to June 2014 showed that, although APRI, F1, AAR, FIB-4, and King's score correlated with the endoscopy results, it was not wise to completely rule out endoscopic findings based on the results of these markers [27].

In a comparative study done in Bangladesh between AAR, APRI, AARPRI, and FIB-4, APRI was found to be the most accurate diagnostic marker among all and, therefore, can be used relative to the other markers as an authentic endoscopy substitute [14]. In a study of patients with alcoholic cirrhosis, the mean FIB-4 score was found to be significantly higher in the esophageal variceal bleeding (EVB) group than in the non-EVB group (8.0 and 3.9, respectively), thereby predicting EVB with a diagnostic accuracy of 63.86% [26]. Contrary to that, in a study conducted between 2015 and 2020 by scanning the online hospital data files of 413 patients in the gastroenterology department, it was found that the FIB-4 and AARPRI were statistically significant and had the most accurate diagnostic values for hepatic fibrosis among all the non-invasive markers [28].

Similarly, our research study included all the patients who had proven cirrhosis based on the ultrasound findings within a certain timeframe. Their endoscopic findings were corroborated after calculating the scores of the non-invasive indexes, such as AAR, APRI, AAPRI, FIB-4, King’s score, and S-index. It was found that only AARPRI and FIB-4 were significant enough to be used for detecting the presence of esophageal varices in a cirrhotic patient. From this result, it can be deduced that the ALT, AST, and platelet count together provide a better understanding of esophageal varices than any other laboratory value; FIB-4 and AARPRI are the only markers that have made use of the ALT, AST, and platelet count together. This can be correlated to a research study conducted by Kothari et al. in central India, which showed that FIB-4 and AARPRI were significantly higher in patients with esophageal varices than those without varices [29]. 

Although this study proves that certain non-invasive markers can be used in resource-limited settings to determine the extent of hepatic fibrosis or cirrhosis correlating to the presence of esophageal varices, more research studies must be done in multiple different settings around the world to determine whether or not the data are viable enough to be implemented in our day-to-day settings. We could not find any evidence about the individual sensitivity and specificity of each marker. 

Limitations

No external funding was used by the researchers. Given the sample size of 100, it was challenging to ascertain the accuracy of the obtained results. Additionally, as the study was conducted at a single hospital in Pakistan, caution is warranted when interpreting the findings due to potential limitations in generalizability.

## Conclusions

In conclusion, it was observed that FIB-4 and AARPRI can be used together as reliable markers to assess the presence or absence of esophageal varices, providing evidence for comparing the degree of liver fibrosis with the presence of esophageal varices. The other four markers, AAR, APRI, S-index, and King’s score, were rendered ineffective as their p-values were not significant enough to provide any inference. This study highlights that simpler, non-invasive means may be the future of identifying esophageal varices, although this is not guaranteed, particularly given the single-hospital, single-country setting of the study, with no discussion on the generalizability of the results.

Further research studies worldwide are necessary to confirm whether these results are globally corroborated and to determine whether implementations should be made on a larger scale based on these data.

## Figures and Tables

**Figure 4:**
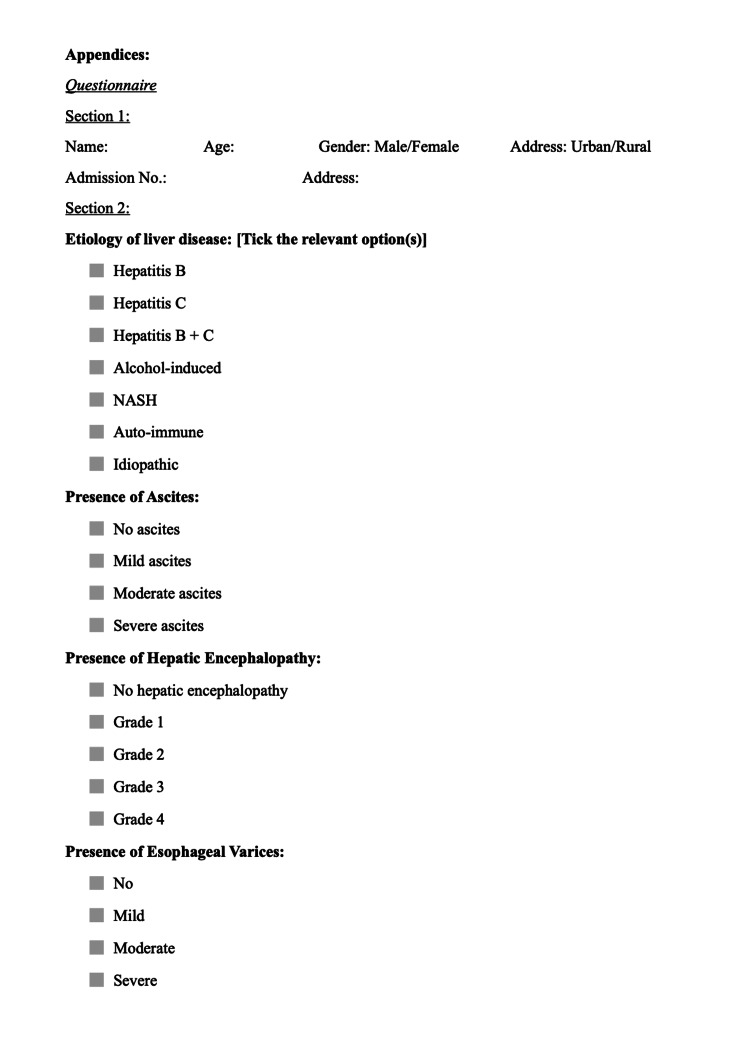
Part one of the questionnaire NASH: non-alcoholic steatohepatitis This questionnaire has been created by the authors of this study

**Figure 5:**
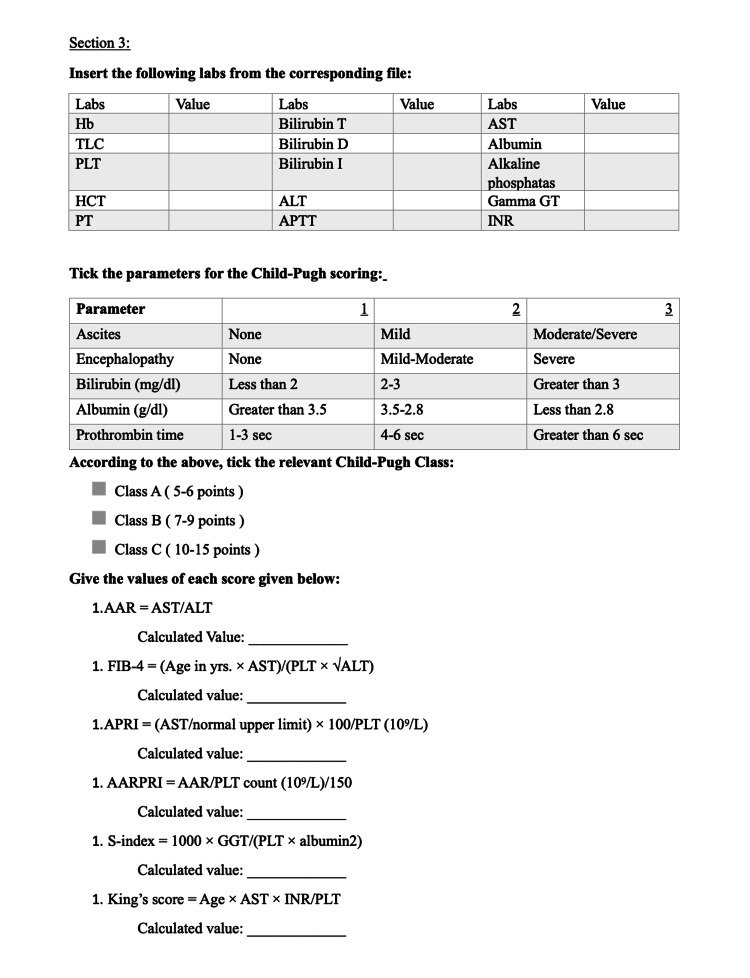
Part two of the questionnaire ALT: alanine transferase; AST: aspartate transferase; PLT: platelet count; GGT: gamma glutamyl transferase; FIB-4: fibrosis-4; AAR: aspartate aminotransferase-to-alanine aminotransferase ratio; APRI: aspartate aminotransferase-to-platelet ratio index; AARPRI: aspartate aminotransferase-to-platelet ratio index; INR: international normalized ratio This questionnaire has been created by the authors of this study

## Appendices

Appendix A

Questionnaire Created for the Study
